# Certified Reference Materials and their need for the diagnosis of autoimmune diseases

**DOI:** 10.31138/mjr.30.1.26

**Published:** 2019-03-28

**Authors:** Evanthia Monogioudi, Ingrid Zegers

**Affiliations:** European Commission, Joint Research Centre (JRC), Geel, Belgium

**Keywords:** Autoantibody measurement, certified reference materials, standardisation, autoimmune diseases

## Abstract

Autoantibody measurement is the chosen tool, in addition to clinical observations, for the diagnosis of autoimmune diseases. Hence, it is essential for these measurements to be reliable and in the longer run to be standardised. Due to the intrinsic variability of analytes and reagents, and the heterogeneity of the available techniques, standardisation cannot be taken for granted, and results may vary between laboratories. As a consequence, diagnoses can be missed or wrong and unnecessary costs may burden individuals and healthcare systems. Standardisation of autoantibody testing is a demanding and multi-parameter task, but could be part of the solution. So as to achieve standardisation, the development and availability of suitable certified reference materials for calibration and/or quality control is crucial.

## AUTOIMMUNE DISEASES AND PREVALENCE

**Figure 1.**
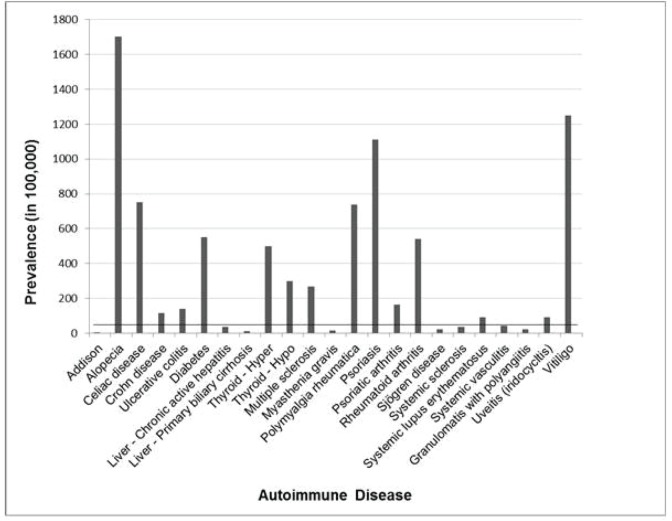
Graphic representation of the prevalence of randomly selected ADs in the USA. The black horizontal line represents the threshold below which a disease is categorised as rare patients in a population of 1000)

Autoimmune diseases (AD) are a cause of concern, not only due to their high and increasing overall prevalence,^1^ but also due to the fact that the costs with which state healthcare systems are burdened are extreme. In 2011, the American Autoimmune Related Diseases Association (AARDA) and the National Coalition of Autoimmune Patient Groups (NCAPG) reported that more than $100 billion are spent yearly for the diagnosis, treatment and care of ADs in the United States.^2^ There are more than 100 diseases currently characterised as autoimmune diseases.^3^ Several groups have used databases and search engines such as Google^1,4^ and MEDLINE^1^ so as to identify incidence and prevalence of various ADs. They are reporting variations in the geographical distribution of ADs as well as between women and men. The observation that many of these reports come to, is that environmental factors seem to be at least as important as genetic factors for the incidence and prevalence of an AD. *Figure 1* presents a graph with some ADs and their prevalence in the USA.^5–15^ As many of them are rather uncommon, difficult to diagnose, and often not reported, capturing them all in epidemiological studies is rather unreliable. Therefore, the prevalence rates presented in this figure are conservative estimates coming from the studies available. Many ADs are rare, but taken together, they affect large numbers of people. In a workshop held in September 2015 in Brussels,^16^ it was summarised that there is an overall increase on the prevalence of autoimmune diseases worldwide for several reasons. It is of the outmost importance to understand ADs, their causes and the relation each disease has to one another, so as to improve diagnosis and disease management. According to the same report -agreeing with many others- the amount of data currently available in regard to ADs is insufficient and tend to focus on specific ADs and in industrialised countries only.^16^ However, a common conclusion is that ADs should not be treated and thought of independently, but rather as a whole. Rarity should not be confused with lack of importance or severity.

### Treatment possibilities

Serological testing is very important for the diagnosis and subsequent treatment choice for a patient. Several ADs, such as small vessel associated vasculitis, consist of subtypes where different markers are responsible for each of them. Therefore, tests able to accurately distinguish and measure those are necessary. ADs are traditionally treated with immunosuppressive medication. Despite their efficiency, when used in long-term treatments, these may cause the patient to be susceptible to infections and malignancies.^17,18^ Additionally, many of these medicines can be toxic and have serious side effects.^18^ For these reasons, the scientific society is turning towards the use of biologics and biosimilars. Biologics are complex molecules created in living cells targeting and inhibiting specific immune cells expected to be involved in ADs.^19^ Biosimilars are biologic products that have demonstrated an action similar to an FDA-approved biologic product. Their only difference to a biologic product is that they may show minor differences is clinically inactive components.^19^ This new generation of medicine –both biologics and biosimilars- is promising, and has already shown to improve treatment outcomes. However, as they do affect the immune responsiveness, they can as well have negative effect on a patient’s health.^17^ At the same time, they are very costly, making them inaccessible to a number of people suffering from an AD. In all cases, whether biologics or traditional therapies are being chosen for the treatment of an AD, it is clear that the right treatment has to be chosen: the most straightforward way to assure that is by ensuring that an accurate and correct diagnosis is made.

## CLINICAL MEASUREMENTS AND UNCERTAINTY

The detection and quantification of autoantibodies is important for clinicians so as to accurately and timely diagnose and treat autoimmune diseases. A variety of ELISA-based immunoassays have been developed throughout the years, which in most cases offer quantitative results. These tests need to be analytically selective and sensitive while at the same time they should preferably show small inter-assay variation. The latter is important for the establishment of common reference ranges and consistent “cut-off” values.^20^ As people are moving from place to place, they demand to have equivalent test results independent from the laboratory where a sample analysis is taking place. This means that different assays are required to give at least comparable analytical results. It is not only a reasonable demand from people that may be unaware of the fact that their test results may vary, but also from legislation and international standards. The International Organization for Standardization has issued several standards describing the requirements for traceability in In-Vitro Diagnostics (ISO/CEN 17511).^21^ In 1998, the EU Directive on In Vitro Diagnostic Medical Devices (IVD-MD) (Directive 98/79/EC) was published, which requires traceability of calibrants and control materials to reference measurement procedures and/or reference materials of higher order.^22^ This high-impact directive (98/79/EC) as well as the Commission Decision 2010/227/EU, will be repealed and replaced in May 2022 by Regulation (EU) 2017/746 of the European Parliament and of the Council of 5 April 2017 on IVDs. Because of these legislations, IVD manufacturers must ensure that the systems they market are properly calibrated against certified reference materials (CRMs) and reference measurement procedures.

The importance of enzyme-linked immunosorbent assays (ELISA) for specific antibodies is also shown in a proposed revision of the 2012 Chapel Hill Consensus for ANCAs (anti-neutrophil cytoplasmic autoantibodies) in small-vessel vasculitis. In the proposal of 2017, the importance of ELISA techniques for the diagnosis of small-vessel vasculitis is highlighted.^23,24^

Accurate detection and quantification of autoantibodies is not ensured despite the high number of good quality assays available in the market. As we have described in previous work^25^ there are a number of factors contributing to the variability of autoantibody tests. These briefly include, but are not limited to, an increased measurement uncertainty due to high coefficient of variability (CV) of the measurements, variability due to calibration issues, the variability in the units used, as well as the nature of antibodies per se. It is important to understand that a good performance from an individual assay does not ensure that the numerical value will be in agreement with that obtained with another assay performing equally well.^26–28^ The lack of result agreement due to the use of different scales by the different assays is shown through several EQAS studies (External Quality Assessment Service), as for example observed for the ANCA distribution in the year 2013.^29^ In this study, negative and positive samples were distributed and analysed by several assays. These assays perform perfectly well independently, but occasionally have an overlap in the value range just when a sample would be classified as positive or negative. This overlap could have as a potential consequence the misinterpretation of a sample’s result and subsequent mistreatment of the patient. Another example where, despite best efforts, solid-phase immunoassays such as ELISA have not managed to be established as the most appropriate method of choice, is the detection of antinuclear antibodies (ANA). Immunoassays such as these would be cheaper and easier to perform than the currently and commonly used indirect immunofluorescence (IIF). However, lack of standardisation has resulted in an increased number of unreliable results and thus in IIF remaining the gold standard for ANA testing.^26^

## CRM EFFORTS WORLDWIDE

The Joint Research Centre (JRC) of the European Commission is an active participant of the Committee on Harmonisation of Autoantibody Testing (WC-HAT) established by the International Federation of Clinical Chemistry and Laboratory Medicine (IFCC) in 2009. The main aim of this committee is to evaluate the most important causes of result variability, to identify the autoantibodies for which a common calibrator would reduce inter-assay variation, and eventually to develop and produce materials that could be used for calibration and quality control. These materials should be certified, with well-defined properties and be commutable, i.e., resemble patient samples.^30^ The proper use of a Certified Reference Material -even though not promised to be the absolute solution to the lack of standardisation- could assist in having more comparable and harmonised measurement results; particularly over time. The JRC has produced, until the moment that this paper is being written, 67 CRMs for health related applications.^31^ These CRMs vary from pure standards and synthetic materials, to matrix materials certified, for example, for their hormone content, their protein content, their total element content and other properties, their catalytic activity, their DNA sequence, and their mass concentration. In addition to the JRC, other organisations like national metrology institutes (e.g., National Institute of Standards and Technology in the USA [NIST]), companies like Cerilliant (a subdivision of Sigma Aldrich), and the National Institute for Biological Standards and Control (NIBSC) from the UK, are also producing Reference Materials (RM) and/or CRMs. Actually, the first initiative in regard to the stan-dardisation of autoantibody testing began already in the 1980s, through a joint effort from several participants including the US Centre for Control and Disease (CDC), the Arthritis Foundation, the International Union of Immunological Societies, and the World Health Organisation (WHO). The result of this collaboration is the storage at the facilities of CDC of sera with monospecific reactivity to a given autoantigen from patients suffering from a systemic autoimmune rheumatic disease (SARD). These samples are available upon request.^26,32^

## ERM-DA476/IFCC AND ERM-DA483/IFCC

**Figure 2.**
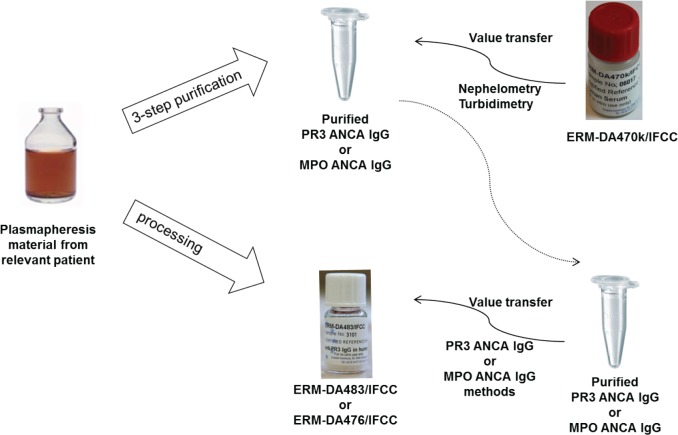
Scheme for the production and characterisation of two CRMs (ERM-DA483/IFCC and ERM-DA476/IFCC) and their relevant calibrants (republished ©EU, 2019).

An example of matrix CRMs produced by the JRC and certified for their specific protein content are ERM-DA476/IFCC^33^ and the ERM-DA483/IFCC.^34^ These two CRMs have been developed for the measurement of antibodies related to the diagnosis of small vessel associated vasculitis. The first one is certified for its mass concentration of immunoglobulin G (IgG) selective for myeloperoxidase (MPO) antineutrophil cytoplasmic antibodies (ANCA) in human serum, while the second one is certified for its proteinase 3 (PR3) ANCA IgG concentration. Both antibodies are seen in ANCA associated vasculitis, which include microscopic polyangiitis (MPA), eosinophilic granulomatosis with polyangiitis (EGPA), and granulomatosis with polyangiitis (GPA).^35,36^ Each of these materials was prepared from a single patient plasmapheresis sample provided by Serum Staten Institute (DK). The specific patient samples were chosen because of their titre of MPO ANCA and PR3 ANCA autoantibodies as well as for the fact that they were shown to be commutable with most of the assays used. They were produced according to ISO Guide 34:2009^37^ and are certified in accordance with ISO Guide 35:2006.^38^ Prior to the development of the CRM, an extensive commutability was performed. Through this study, the best format for the CRM was chosen from all those tested (plasma or serum, lyophilised or liquid-frozen, different raw materials, different concentrations and/or buffer solutions). After this preliminary study was completed, and the commutability of both the matrix material and the purified IgG autoantibody was verified, the serum material was aliquoted into glass vials, closed with rubber stoppers and screw caps under a noble gas atmosphere such as Argon, and stored at −70 °C. The materials were then studied for their homogeneity both between and within a selected number of vials (which corresponds to approximately the cubic root of the total number of units produced), as well as for their stability during transport for a period of up to one month, for three different temperatures and for their long term stability (up to 2 years) at two different temperatures. The next step in the characterisation of CRM such as the ones mentioned, was to define what was the mass concentration of the protein of interest in a unit traceable to the SI (specifically, in these two examples in mg/L). For this purpose, a minimum of six assays was employed, and each participant laboratory was required to use their own methodology and instrumentation so as to negate laboratory bias and reduce the combined uncertainty of the measurements. Measurements of several dilutions and independent replicates took place on four separate days and the results were submitted by the participating laboratories to JRC to perform the necessary calculations. No values were disregarded on statistical grounds, only ensuring that all results -unless technically invalid- were taken into account for the final calculation of the mass concentration of the protein of interest. The actual value assignment was made following a value transfer procedure as described by Blirup-Jensen in 2008.^39^ In this procedure, the first step was production and characterisation of a preparation of the protein of interest (i.e., MPO ANCA IgG and PR3 ANCA IgG respectively) in a purified form. It was verified that this solution contains only IgG selective for MPO or PR3, respectively. This pure protein preparation was used for preparing calibration solutions for the (immunoassay) measurements for characterisation of the CRMs. The characterisation and value assignment of the pure protein preparation was made by determining the total IgG content, as all the IgG in the solution had the required selectivity. These measurements were performed by laboratories by commonly used clinical assays for IgG, such as turbidimetry and/or nephelometry. They measured both the purified preparation of the protein of interest (i.e., MPO ANCA IgG and PR3 ANCA IgG respectively) and vials of another material which has a known concentration of total IgG (ERM-DA470k/IFCC),^40^ and values for the specific IgG preparations were derived from the ratio of the results. The detailed protocols for the procedure followed for the development for each material as well as the assays used for the characterisation can be found in the relevant publications.^33,34^ *Figure 2* depicts a republished graphic representation of the steps for the development of the two CRMs (this figure can also be found at the certification report of the material ERM-DA483/IFCC).^34^

The main purpose of the CRMs is to be used in the calibration of immunoassay-based in vitro diagnostics devices and/or as calibrators. *Table 1* shows the results reported from the ten assays used in the characterisation study of the ERM-DA483/IFCC material. In this table, all results presented are of the same material (the candidate CRM). It can be clearly seen that results are varying, not only -as expected- when they are reported in different units, but variations do exist between results reported in the same, but arbitrary, unit (*Table 1*). In 2016, Monogioudi et al. published a paper where results are graphically represented from the recalibration of the measured values for the CRM, ERM-DA476/IFCC using the responses for the purified MPO ANCA IgG preparation for each assay.^27^ In the published figure, it is shown that result variation was reduced to 13% through the use of a common calibrant, while at the same time, the results were reported in the same SI unit (mg/L) and not in the arbitrary units used by each assay that make direct comparison impossible. A similar figure with the impact of recalibration of results for the ERM-DA483/IFCC material was submitted in October 2018 elsewhere.

**Table 1.** Methods used for the characterisation of the ERM-DA483/IFCC material.

**Method Name**	**Principle**	**Concentration [reported units]**	**Concentration [mg/L]**
ImmuLisa™ Proteinase 3 (PR3) antibody Enhanced	ELISA	329.78 IU/mL	333.63
ORG 618 PR3 hs	ELISA	153.43 U/mL	319.75
BioPlex 2200 Vasculitis	Multiplex Flow Immunoassay	3.15 AI	252.95
Anti-PR3 EIA	ELISA	95.18 AI	247.58
QUANTA Lite PR 3	ELISA	331.96 U	216.84
QUANTA Flash	Chemiluminescent immunoassay	255.43 CU	255.01
PR3 ANCA Wieslab®	ELISA	78.68 IU/mL	258.59
AESKULISA PR3 sensitive	ELISA	364.74 U/mL	238.77
EliA PR3^s^	Fluorescence immunoassay	105.22 IU/mL	275.33
Anti-PR3-hn-hr-ELISA (IgG)	ELISA	348.32 RU	301.77

All methods used for the characterisation of the ERM-DA483/IFCC material. The principle its method is representing, as well the concentration of PR3 ANCA IgG before correction and after correction using the certified value of the CRM, are given. The units on the third column are the ones given in each assay, while on the fourth column, all values are in mg/L. (SI)

IU/mL: international units / mL; AI: antibody index values; CU: chemiluminescent units; RU: relative units

## CONCLUSION

The JRC of the European Commission is continuing its close collaboration with the IFCC for setting up new standardisation targets and defining new goals. The current approach of using antigen-affinity-purified immunoglobulin fractions or fully characterized monoclonal antibody preparations has so far shown to be a successful approach.

At this moment the development of a material certified for its anti-beta 2 glycoprotein IgG content is in progress, while preparation steps for a CRM against anti glomerular basement protein IgG have started. Measurements of autoantibodies by ELISA-type assays are nowadays required for the diagnosis of ADs. However, despite their widespread use, the variability of these assays often causes discrepancies between reported results, which have as consequence a possible misdiagnosis and impact the usefulness of the chosen treatment. Therefore, standardisation of the available assays is important. In order to achieve that, in addition to the guidelines and legislation developed, availability and use of suitable CRMs for calibration and/or quality control is necessary despite being complicated and often difficult. A CRM cannot solve entirely the problem of assay harmonisation; however, an appropriate and well characterised CRM can reduce inter- and intra-assay variability and retain scales stable over time. The standardisation of protein biomarkers is possible provided that everyone involved, whether in standardisation, calibration or simply use of the assays, is aware of potential pitfalls and difficulties, such as the nature of the antigens used in an assay. For example, clarification of the epitopes that are reactive in a particular assay would shed light in regard to what is actually measured. It is without doubt a very demanding task. Unfortunately, due to the nature of protein biomarkers, there are hardly any generally applicable recipes for standardisation and CRM production. However, the efforts taking place worldwide not only for standardisation but also for increasing awareness of the problem, are good steps forward.

Finally it is important to stress that at the moment the funding allocated to autoimmune diseases does not necessarily reflect on the importance of these diseases (for example in 2014/15, only 5.5% of the UK’s Department of Health’s National Institute for Health Research (NIHR) was allocated to inflammatory and immune diseases).^41^

Often, autoimmune diseases are being considered as single entities, and that partially explains the lack of funding. If they are taken individually, they are rather rare, thus affecting only a small number of the population. However, it is important to understand that ADs share more than enough similarities so as to be considered as one entity especially in regard to the research, funding and efforts put on them. On the whole, ADs affect a very large part of the population worldwide, and as a consequence, entail a great cost for healthcare systems and individuals.
